# Silkworm Hemolymph and Cocoon Metabolomics Reveals Valine Improves Feed Efficiency of Silkworm Artificial Diet

**DOI:** 10.3390/insects15040291

**Published:** 2024-04-19

**Authors:** Jinxin Wu, Lingyi Li, Daoyuan Qin, Han Chen, Yuanlin Liu, Guanwang Shen, Ping Zhao

**Affiliations:** 1 Integrative Science Center of Germplasm Creation in Western China (Chongqing) Science City, Biological Science Research Center, Southwest University, Chongqing 400715, China; 2 Westa College, Southwest University, Chongqing 400715, China

**Keywords:** silkworm, valine, artificial diet, mulberry leaf, feed efficiency

## Abstract

**Simple Summary:**

The silkworm is an economically important insect, and the popularity of artificial feed has promoted the development of the sericulture industry. However, the improvement of feed efficiency remains a challenge. This study used metabolomics-based methods to analyze the differences in silkworm cocoons and hemolymph between the mulberry-leaf group and the artificial-feed group. It was found that the concentration of amino acids was significantly higher in the mulberry-leaf group than in the artificial-diet group. In a comparison of the effects of different concentrations of valine on silkworms fed the artificial diet, it was found that the food intake of the 2% and 4% valine groups was significantly lower than that of the 0% valine group, but the cocoon-production efficiency was significantly improved. This study demonstrates that valine is an important amino acid that can regulate feed efficiency, providing a new direction for the improvement of artificial feed.

**Abstract:**

Artificial silkworm diets significantly impact farm profitability. Sustainable cocoon production depends on the continuous improvement of feed efficiency to reduce costs and nutrient losses in the feed. This study used metabolomics to explore the differences in silkworm cocoons and hemolymph under two modes of rearing: an artificial diet and a mulberry-leaf diet. Nine metabolites of silkworm cocoons and hemolymph in the mulberry-leaf group were higher than those in the artificial-diet group. Enrichment analysis of the KEGG pathways for these metabolites revealed that they were mainly enriched in the valine, leucine, and isoleucine biosynthesis and degradation pathways. Hence, the artificial silkworm diet was supplemented various concentrations of valine were supplemented to with the aim of examining the impact of valine on their feeding and digestion of the artificial diet. The results indicated that valine addition had no significant effect on feed digestibility in the fifth-instar silkworm. Food intake in the 2% and 4% valine groups was significantly lower than that in the 0% valine group. However, the 2% and 4% valine groups showed significantly improved cocoon-production efficiency, at 11.3% and 25.1% higher, respectively. However, the cocoon-layer-production efficiencies of the 2% and 4% valine groups decreased by 7.7% and 13.9%, respectively. The research confirmed that valine is an effective substance for enhancing the feed efficiency of silkworms.

## 1. Introduction

Silkworms are economically important insects with well-characterized genetics [1]. Basic research is moving towards model biology and the mechanization of artificial-diet-based breeding. Feed efficiency is an important indicator in large-scale animal production. Feed efficiency can be expressed as “the amount of feed ingested”/“product output”. For silkworms, the most-studied metrics are “leaf–silk transformation rate” [2,3] and “full cocoon-layer-production efficiency” [3,4]. Most studies on the “leaf–silk transformation rate” have used mulberry leaves as the silkworm food, and studies on the feed efficiency of artificial-diet-reared silkworms are relatively limited.

Metabolomics can reflect specific metabolic characteristics of an organism by identifying and quantifying the metabolites in certain tissues or biological fluids at specific time points [5]. An increasing number of -omics applications are being used to study the physiological responses of silkworms to different diets. Wu et al. conducted metabolomic analysis of the midgut, hemolymph, and the posterior silk gland of silkworms fed an artificial diet and identified six biomarkers associated with cocoon yield [6]. Dong et al. compared the metabolic differences between the hemolymphs of silkworms reared on mulberry leaves and an artificial diet and found that silkworms fed the artificial diet exhibited disorders of amino acid metabolism and downregulation of carbohydrate metabolism, energy metabolism, and lipid metabolism [7]. Tao et al. compared the metabolic differences among the hemolymphs of silkworms reared on different artificial diets and confirmed significant changes in the metabolism of amino acids, uric acid, carbohydrates, lipids, and vitamins in the hemolymph of silkworms reared on an artificial diet throughout fifth instar stages of development [8]. Li et al. compared the differences in midgut metabolites between silkworms reared on mulberry leaves and artificial diet, revealing differences in physiological functions such as disease resistance and immunity, silk quality, and growth and development of silkworms fed the artificial diet [9]. Previously, we studied the metabolomics of excrement from silkworms reared on mulberry-leaf and artificial diets. We found that the relative contents of amino acids, carbohydrates, and lipids in excrement from silkworms reared on artificial diets decreased significantly, whereas the relative contents of urea, citric acid, and other organic acids increased significantly [10].

Cocoons are important products of silkworms, and the hemolymph is the fluid tissue for material exchange in the circulatory system. Metabolic changes in the silkworm hemolymph can greatly affect silk production. Feeding silkworms with low concentrations of silver nanoparticles can increase the weight of the cocoons [11]. Researchers have also increased the weight of the cocoon shells by injecting silkworms with titanium dioxide nanoparticles [12]. By comparing the metabolic differences between silkworms with blocked silk synthesis, Chen et al. observed that increasing the dose of the glycine injection increased the weight of the cocoon layer of silkworms [13]. Furthermore, by comparing differences in amino acid metabolism between silkworms reared on a mulberry-leaf diet and an artificial diet, researchers found that adding glycine to an artificial diet could improve the rate of cocoon-shell production [14]. This finding indicates that the different nutritional components of artificial feeds have a significant impact on the metabolism of the hemolymph of silkworms. However, studies that examine both the changes in the hemolymph metabolites and the changes in the final cocoon are limited. Therefore, we speculate that research on metabolites that considers both the hemolymph and the cocoon in silkworms reared on different feedstuffs may provide a reference for the improvement of artificial feed.

In this study, we determined that valine is the key amino acid that affects the feed efficiency of the silkworm. We made this finding by jointly analyzing the changes in metabolites in the silkworm cocoon and the hemolymph; the results showed that valine improves the conversion efficiency of the silkworm.

## 2. Materials and Methods

### 2.1. Experimental Insects and Diets

Individuals of the silkworm strain Liangguang II were obtained from our laboratory at Southwest University, Beibei, China. They were reared on either an artificial diet or on fresh mulberry leaves supplied by Southwest University. The mulberry leaves were the Chinese ‘JiaLing20’ variety and were cultivated in a field at Southwest University. The silkworms were grown from hatching until cocoon formation at 25 °C under a 12 h light/12 h dark photoperiod. The feed formula was the same as that used in a previous study: 30% defatted soybean powder, 25% mulberry-leaf powder, 19.6% starch, 10% cellulose powder, 8% agar, 2% vitamin C, 2% citric acid, 1.5% vitamin B complex, 1% mineral salts complex, 0.5% potassium sorbate, 0.2% choline chloride, and 0.2% calcium propionate [10].

### 2.2. Cocoon and Hemolymph Samples Collection

Hemolymph from silkworms reared on mulberry leaves and silkworms reared on an artificial diet were collected on day 5 of the fifth instar. The collection was repeated for eight tubes of samples for each pooled sample of hemolymph (each tube of samples contained hemolymph from three silkworms) and stored at −80 °C for later use after quick freezing in liquid nitrogen. Cocoons were collected 7 days after clustering for the experiment.

### 2.3. Feeding with Different Concentrations of Methionine

To prepare the feed, 0 g, 0.5 g, 2 g, and 4 g of methionine were separately added to four 100 g samples of dry feed powder. After thorough mixing with water, the mixture was steam-cooked for 30 min to prepare artificial diets containing 0%, 0.5%, 2%, and 4% methionine. These artificial diets with different concentrations of methionine were fed to silkworms (*n* = 30), and the daily body weight of the silkworms during the fifth instar stage (*n* = 10) was recorded. At the fifth instar stage, the daily feed amount was recorded, and after 24 h, the remaining food and dry weight of excrement were separately weighed to calculate the intake, digestion, and digestion rate per silkworm (*n* = 10).

### 2.4. Measurement of Developmental Parameters

Cocoon-production statistics included cocoon weight, cocoon shell weight, and cocoon–shell ratio (%). Cocoon weight (*n* = 30), cocoon shell weight (*n* = 30), and cocoon–shell ratio (%) [(cocoon shell weight/cocoon weight) × 100%] were recorded on day 6 of the pupal stage. Outcomes were calculated as follows: cocoon-production efficiency (%) = total cocoon weight (g)/total dry-matter intake at the fifth instar (g) × 100%; production efficiency of the cocoon layer (%) = cocoon-layer weight (g)/total dry matter intake at the fifth instar (g) × 100%; dry-matter digestion = dry-matter ingestion − dry-matter defecation; dry-matter ingestion amount = feeding amount − residual bait amount; digestibility (%) = dry matter digestion/dry matter ingestion × 100%. Data were expressed as mean ± SEM. Differences in data between silkworm subjects were assessed using Student’s *t*-test (two-tailed, two-sample equal variance hypothesis).

### 2.5. GC-MS Sample Pretreatment

The sample processing steps for the hemolymph were as follows [15]:

Hemolymph samples were placed on ice until they thawed. The tubes were centrifuged (5000× *g* for 10 min at 4 °C). A sample (80 μL) was taken, and 320 μL methanol (without an internal standard) was added. The mixture was vortexed for 2 min and placed on ice for 1 h. Centrifugation was performed at 14,000× *g* for 10 min at 4 °C, and 300 μL supernatant was collected. The precipitate obtained by low-temperature freeze-drying was stored at −80 °C for future use.

The sample-processing steps for the cocoon were as follows [16]:

The clean cocoons were weighed (0.01 g) and treated in 100 °C 1 mL water for 30 min. After centrifugation (14,000× *g*), the supernatant (800 μL) solution was transferred into a new centrifuge tube and lyophilized.

The derivation steps were as follows [10]:

(1) Freeze-dried samples of silkworm cocoons and hemolymph were redissolved in 80 μL methoxyamine solution (20 mg/mL).
(2) The sample was vortexed for 2 min and treated with ultrasonic wave for 15 min after instant separation. Derivatization was carried out at 37 °C for 1.5 h.
(3) MSTFA (60 μL) was added with instantaneous centrifugation. The vortex instantaneously dissociated at 37 °C for 1 h.
(4) After immediate separation, 10 μL n-heptane was added. The mixture was vortexed, which resulted in transient dissociation.
(5) Centrifugation was performed at 14,000× *g* for 20 min at 10 °C.
(6) The supernatant (100 μL) was sucked into the loading bottle.

Analysis conditions of GC-MS

Analysis was conducted on an Agilent 7890B-5977A system (Agilent Technologies, Santa Clara, CA, USA) with a DB-5MS column (0.25 μm × 0.25 mm × 30 m). The injected volume was 1 μL, and the split ratio was 10:1. The sample inlet temperature was 300 °C; the transmission temperature was set to 280 °C; and the ion source was set to 230 °C. The heating program included maintaining the initial 70 °C for 3 min, then increasing it to 170 °C at 5 °C min^−1^, 234 °C at 4 °C min^−1^, 270 °C at 5 °C min^−1^, and 300 °C at 10 °C min^−1^, with the final temperature maintained for 5 min. The detector voltage was set to 0.93 kV. The metabolite EI voltage was set to 70 eV. Full-scan mode (*m*/*z*: 33–600) was used to collect high-quality signals.

### 2.6. GC-MS Data Processing

The original GC-MS mass spectrum file was converted to an AIA data format file, and XCMS processing was used (https://xcmsonline.scripps.edu/index.php, accessed on 17 March 2024). The metabolites were identified by comparing their mass spectra and retention indices with reference data from the National Institute of Standards and Technology (NIST, Gaithersburg, MD, USA). The metabolites from the mulberry-leaf and artificial-feed groups were confirmed if they had a retention match value greater than 0. PCA was conducted using the website http://v2.biodeep.cn/home accessed on 17 March 2024 to identify differences in metabolites between the groups. The differences in metabolite content between silkworm cocoons and hemolymph were analyzed using Student’s *t*-test (double-tailed, double sample, and equal variance hypothesis) in Microsoft Excel 2016 (Microsoft Corp., Redmond, WA, USA). The KEGG database was obtained from The KEGG website (http://www.kegg.jp/ accessed on 17 March 2024). Another website (http://v2.biodeep.cn/home accessed on 17 March 2024) was used to generate a heat map and conduct a z-score analysis and KEGG enrichment analysis.

## 3. Results

### 3.1. Cocoon and Hemolymph Extract Metabolic Profiles of Silkworms Reared on Fresh Mulberry Leaf and Artificial Diet

Table 1 lists the metabolites that showed significant differences between the mulberry-leaf-group silk (MC) and the artificial-diet-group silk (AC) (*p* < 0.05). The z-score and a heat map based on the abundance of identified differential metabolites are shown in Figures S1A and S2A, respectively. A total of 22 differential metabolites were identified (*p* < 0.05). Compared to the silkworm cocoons in the artificial-diet group, 12 metabolites were upregulated and 10 metabolites were downregulated in the silkworm cocoons in the mulberry-leaf group. In the mulberry-leaf group, the abundance of metabolites in silkworm cocoons was higher than that in the artificial-diet group, and the top 10 metabolites were, in descending order of abundance, *L*-asparagine, serine, glycine, *L*-valine, *L*-isoleucine, *L*-leucine, *L*-threonine, phenylalanine, 2-butenedioic acid(E), and *L*-tyrosine. Therefore, amino acids were the main metabolites present at high levels in the silkworm cocoons of the mulberry-leaf group. In the artificial-diet group, the metabolite content in the silkworm cocoons was higher than that in the mulberry-leaf group, and the top 10 metabolites were urea, *D*-pinitol, glycerol, *D*-gluconic acid, (R*, S*)-3,4-dihydroxybutyric acid, benzoic acid, propanoic acid, 2,3,4-dihydroxybutyric acid, D-(+)-talose, and butanedioic acid.

Table 2 lists the hemolymph metabolites with significant differences between the hemolymphs of the mulberry-leaf group (MH) and the artificial-diet group (AH) (*p* < 0.05). The Z-score and heat map based on the abundance of the identified differential metabolites are shown in Figures S1B and S2B, respectively. A total of 38 differential metabolites were identified (*p* < 0.05). Among them, compared with the hemolymph of the artificial-diet group, 22 metabolites were upregulated and 16 metabolites were downregulated in the hemolymph of the mulberry-leaf group. The metabolite abundance in MH was higher than that of AH, and the top 10 metabolites were *D*-glucopyranoside, *L*-valine, *L*-leucine, butyric acid, 2-keto-L-gluconic acid, m*yo*-inositol, *L*-isoleucine, methylmaleic acid, *DL*-arabinose, 2-hydroxymandelic acid, and *L*-threonine. Therefore, amino and organic acids were the main metabolites with high content in MH. The content of metabolites in AH was higher than that of MH, and the top 10 metabolites were pantothenic acid, aminomalonic acid, mannitol, ritalinic acid, pentanedioic acid, *D*-sorbitol, urea, *N*, *N*-dimethylglycine, β-alanine, and *D*-trehalose.

**Figure 1:**
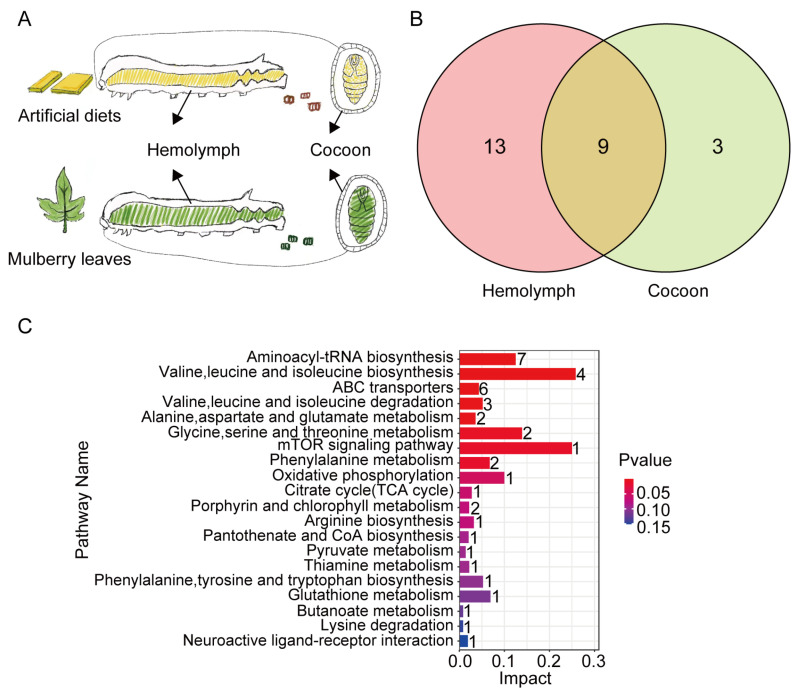
Cocoon and hemolymph metabolites were present at higher levels in the mulberry-leaf group than in the artificial-diet group. (**A**) Analysis diagram. (**B**) Venn diagram. (**C**) KEGG enrichment pathway. The abscissa of the metabolic pathway map (pathway impact) is calculated based on topology analysis. The numerical value indicates the importance of the metabolic pathway in the overall metabolic network. The greater the numerical value, the more important the pathway is. The ordinate represents the enriched metabolic pathway, and the value on the pathway represents the total number of metabolites in the target metabolic pathway.

The cocoons and hemolymph of silkworms reared on mulberry leaves and an artificial diet were analyzed jointly (Figure 1A). Twelve silkworm cocoon metabolites were observed to be present at higher concentrations in the mulberry-leaf group than in the artificial-diet group. Similarly, 22 metabolites that were found at higher levels in the silkworm hemolymph from the mulberry-leaf group than in the hemolymph from the artificial-diet group, with 9 metabolites consistently differing in both the cocoon and hemolymph (Figure 1B). Through KEGG enrichment analysis of these 9 metabolites, we found that the different metabolites were primarily enriched in valine, leucine, and isoleucine biosynthesis and in valine, leucine, and isoleucine degradation (Figure 1C). Therefore, we speculated that valine may be an important amino acid that regulates silkworm pupa balance and selected valine for the next feeding experiment.

### 3.2. Effect of Valine on Feed Efficiency of Silkworm, Bombyx Mori

**Figure 2:**
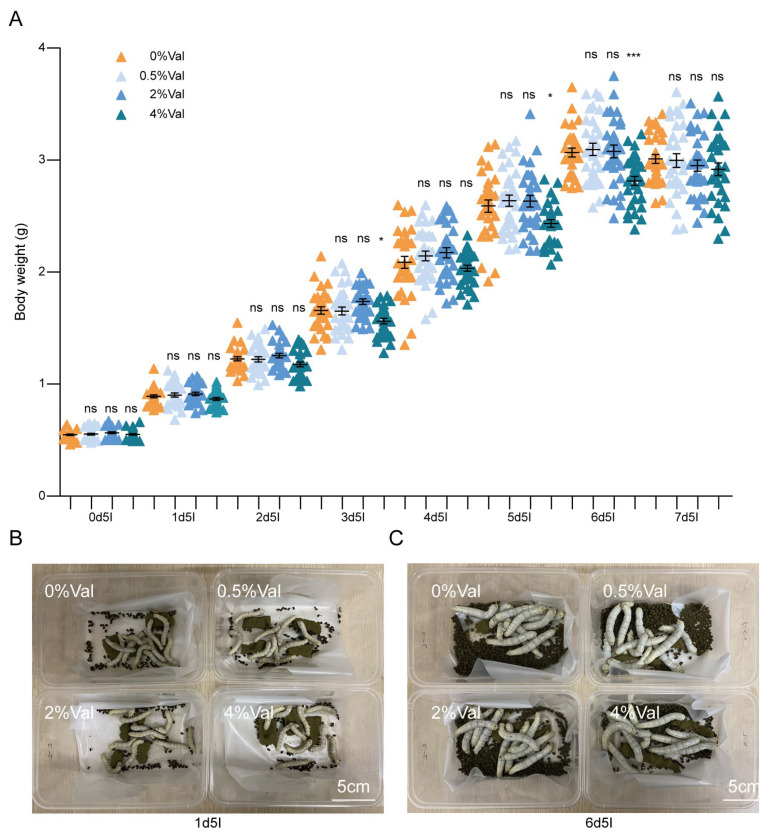
The effect of valine on silkworms. (**A**) The weight of the silkworms. (**B**,**C**) The bodies of the silkworms on the first day of the fifth instars and day 6 of the fifth instar, respectively. Silkworm ages and time of silkworm growth and development: the first number represents the date, and the second number represents the age (for example, 0d5l represents day 0 of the fifth instars). 0%Val: 0 g valine added to 100 g dry diet powder; 0.5%Val: 0.5 g valine added to 100 g dry diet powder; 2% Val: 2 g valine added to 100 g dry diet powder; and 4% Val: 4 g valine added to 100 g dry diet powder. Thirty silkworms were in each experimental group. Data were expressed as mean ± SEM. Differences in data were assessed by Student’s *t*-test (two-tailed, two-sample equal variance hypothesis), * *p* < 0.05; *** *p* < 0.001; ns *p* > 0.05.

Because differences were observed between male and female individuals, fifth-instar males were selected for daily detailed investigation [17,18,19]. Silkworms fed the different diets are shown in Figure 2B,C. From the fifth instar, no significant difference was observed in the weight of the silkworms fed different concentrations of valine before the fifth day of the 4th instar. However, the weight of the 4% valine group was significantly lower than that of the other groups on days 5 and 6 of the fifth instar (Figure 2A). The weights of silkworms in the 0%, 0.5%, and 2% valine groups reached a peak or maximum value on day 6 of the fifth instar, whereas the weight of the 4% valine group reached a maximum on day 7 of the fifth instar.

**Figure 3:**
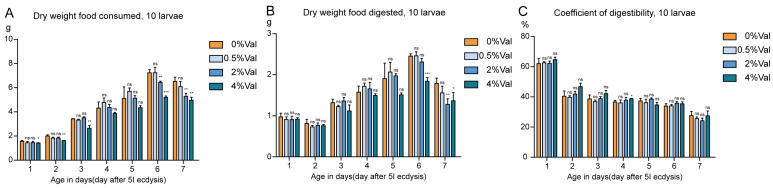
The feeding ability of silkworms fed a valine diet at the fifth instar. (**A**) Dry weight of food consumed by silkworms; (**B**) Dry weight of food digested by silkworms; (**C**) The coefficient of digestibility; 0%Val: 0 g valine added to 100 g dry diet powder; 0.5%Val: 0.5 g valine added to 100 g dry diet powder; 2% Val: 2 g valine added to 100 g dry diet powder; and 4% Val: 4 g valine added to 100 g dry diet powder. Ten silkworms were in each experimental group, with three replicates in total. Data were expressed as mean ± SEM. Differences in data were assessed by Student’s *t*-test (two-tailed, two-sample equal variance hypothesis), * *p* < 0.05; ** *p* < 0.01; *** *p* < 0.001; ns *p* > 0.05.

The amount of feed added to four types of substances at different concentrations was investigated daily. The dry matter intake of each group increased with age and reached a maximum on day 6 of the fifth instar. No significant difference was observed between the low-concentration (0.5% valine) group and the 0% valine group in terms of silkworm intake of artificial feed containing valine. The dry-matter intake of silkworms in the 4% valine group was lower than that of the control group on the first day of the fifth instar, and the difference between days 6 and 7 of the fifth instar widened further. In the 2% valine group, food intake was lower than in the control group on days 6 and 7 of the fifth instar (Figure 3A,B).

The feed digestibility across groups was not significantly different in the fifth instar, indicating that the addition of valine did not affect silkworm digestion (Figure 3C).

**Figure 4:**
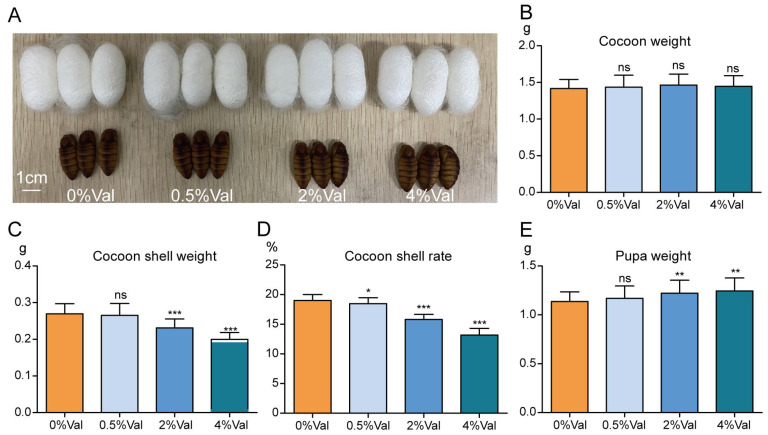
Effects of valine addition in the diet on economic properties of cocoons. (**A**) The cocoon and pupa on day 6 after wandering; The cocoon weight (**B**), cocoon-shell weight (**C**), cocoon-shell rate (**D**) and pupal weight (**E**) of the silkworms; 0%Val: 0 g valine added to 100 g dry diet powder; 0.5%Val: 0.5 g valine added to 100 g dry diet powder; 2% Val: 2 g valine added to 100 g dry diet powder; and 4% Val: 4 g valine added to 100 g dry diet powder. Ten silkworms were in each experimental group, with three replicates in total. Data were expressed as mean ± SEM. Differences in data were assessed by Student’s *t*-test (two-tailed, two-sample equal variance hypothesis), * *p* < 0.05; ** *p* < 0.01; *** *p* < 0.001; ns *p* > 0.05.

Subsequently, we investigated the economic properties of silkworm cocoons. The addition of valine did not affect the appearance of the silkworm cocoons (Figure 4A). No significant differences were observed in cocoon weight among the four groups (Figure 4B). The cocoon-layer weights of the four groups were 0.27, 0.27, 0.23, and 0.19 g, respectively. The addition of 0.5% valine did not affect the cocoon-layer weight, whereas the 2% and 4% valine groups showed decreases of 14.8% and 29.6%, respectively, compared with the 0% valine group, indicating that the high concentration of valine inhibited silk synthesis (Figure 4C). The cocoon-layer rates in the four groups were 19.02%, 18.48%, 15.81%, and 13.17%, respectively. The cocoon-layer formation rate decreased with the addition of valine. The cocoon-layer rates of the 2% and 4% valine groups were 16.9% and 30.8% lower, respectively, than those of the 0% valine group (Figure 4D). The pupal weights of the four groups were 1.14, 1.17, 1.22, and 1.24 g, respectively. The pupal weights of the 2% and 4% valine groups were 7% and 8.8% higher, respectively, than those of the 0% valine group (Figure 4E).

**Figure 5:**
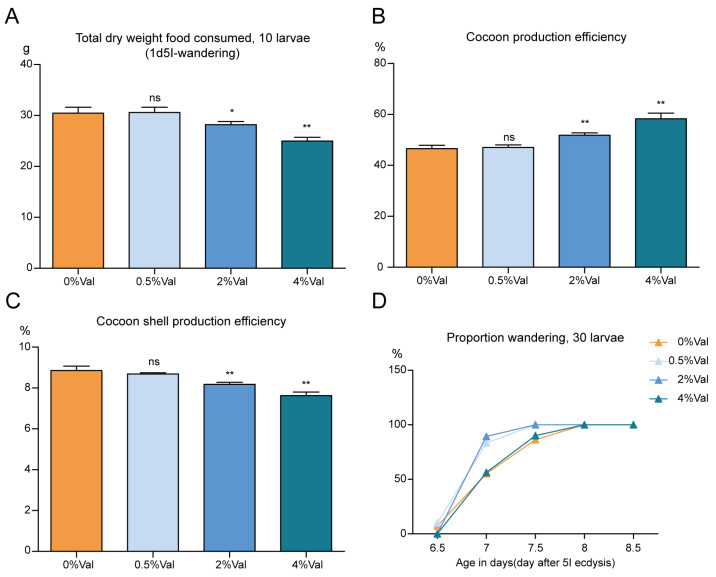
Total food intake and development time of silkworms in the fifth instar. (**A**) The total dry weight of food consumed from 1d5l to wandering (*n* = 10). (**B**) Cocoon-production efficiency; (**C**) Cocoon-shell-production efficiency. (**D**) The fifth-instar development time of silkworms in four groups (*n* = 30). 0%Val: 0 g valine added to 100 g dry diet powder; 0.5%Val: 0.5 g valine added to 100 g dry diet powder; 2% Val: 2 g valine added to 100 g dry diet powder; and 4% Val: 4 g valine added to 100 g dry diet powder. Data were expressed as mean ± SEM. Differences in data were assessed by Student’s *t*-test (two-tailed, two-sample equal variance hypothesis), * *p* < 0.05; ** *p* < 0.01; ns *p* > 0.05.

The total food intake (calculated from the total amount consumed by 10 silkworms) for the entire fifth instar was 7.5% and 18% lower in the 2% and 4% valine groups, respectively, than in the 0% valine group. However, no significant difference was observed in the total food intake of the fifth-instar larvae between the 0% and 0.5% valine groups (Figure 5A). This shows that the feeding amount of silkworms decreases with increasing valine concentration in the feed and that a high valine concentration inhibits the feeding of silkworms. The overall cocoon-production efficiencies of the 0%, 0.5%, 2%, and 4% valine groups were 46.57%, 47.02%, 51.81%, and 58.28%, respectively. The cocoon-production efficiency was not affected by the addition of 0.5% valine. In contrast, the overall cocoon-production efficiencies of the 2% and 4% valine groups were 11.3% and 25.1% higher than that of the 0% valine group, respectively (Figure 5B). The cocoon-layer-production efficiencies of the four groups were 8.86%, 8.68%, 8.18%, and 7.63%, respectively. In contrast, those of the 2% and 4% valine groups were 7.7% and 13.9% lower, respectively, than those of the 0% valine group (Figure 5C). The fifth-instar development times of the 0.5% and 2% valine groups were slightly faster than that of the 0% valine group. In contrast, the difference between the 4% and 0% valine groups was small, indicating that the addition of a small amount of valine tended to enable the silkworms to cluster ahead of time (Figure 5D).

## 4. Discussion

Amino acids are both protein building blocks and signaling molecules. They regulate food intake, gene expression, protein phosphorylation, and intercellular communication [20]. The amino acid content can also affect the synthesis of silk fibroin [6,10,13]. Glycine enhances the conversion efficiency of amino acids to silk fibroin in the silk glands, thereby increasing silk fibroin production [14]. In this study, the levels of L-valine, leucine, isoleucine, threonine, alanine, glycine, phenylalanine, serine, and proline in the hemolymph of silkworms fed the artificial diet were lower than those in the hemolymph of silkworms in the mulberry-leaf group. This difference may explain why silk cocoons produced by silkworms fed artificial feed tended to have lower weights than cocoons produced by those fed mulberry leaves [6,8,21]. At the same time, we also found that levels of L-asparagine, serine, glycine, L-valine, L-isoleucine, L-leucine, L-threonine, phenylalanine, 2-butenedioic acid(E), and L-tyrosine were also lower in cocoons produced by silkworms raised on the artificial diet than in cocoons produced by silkworms in the mulberry-leaf group. Another study reported that L-glutamic acid, L-alanine, and L-aspartic acid promote food consumption in Drosophila in a dose-dependent manner [20]. Therefore, we speculate that the addition of amino acids to the artificial diet can improve the feeding performance of silkworm larvae.

A KEGG pathway-enrichment analysis of differential metabolites in the hemolymph and silk glands of silkworms revealed that the main enrichment pathways were those related to the biosynthesis of valine, leucine, and isoleucine, as well as the degradation pathways of valine, serine, and isoleucine (Figure 1C). Valine is one of three branched-chain amino acids and is an essential amino acid in silkworms. Research has shown that adding 1–2% valine to mulberry leaves can slightly increase the strength and toughness of silk [22]. However, the content of L-valine in the excrement of the silkworms in the mulberry-leaf group was 3.84 times higher than that in the excrement of the silkworms in the artificial-diet group [10]. Additionally, it was found that the L-valine content in the hemolymph of mulberry leaf-fed silkworms was 12.87 times higher than that in the hemolymph of silkworms in the artificial-diet group (Table 2). Studies have reported that the content of valine in mulberry leaves is around 6.63% [14]. The main components of most commercial artificial silkworm diets include mulberry-leaf powder, soybean meal, cornmeal, inorganic salts, and a vitamin mixture, without the separate addition of valine. Therefore, we chose to supplement the artificial diet of silkworms with valine to observe the effects of valine on silkworms reared on an artificial diet.

Valine plays an important role in the feeding process of animals. Rats’ intake of feed lacking valine decreased, and when valine was restored to the feed, their intake returned to normal [23]. Adding valine to low-protein feed can increase the average daily feed intake and average daily weight gain of pigs and reduce the urea nitrogen content in their blood [24]. Within a certain concentration range, the addition of valine to the feed of weanling piglets increases the average daily food intake, and the average daily protein intake also increases with increased valine concentrations. However, when the amount of added valine is too high, the average daily food intake is reduced [25]. We fed domestic silkworms with artificial feed containing different concentrations of valine and found that adding a low dose of valine (≤0.5%) did not affect the economic properties of the silkworm cocoons and that the cocoon-shell-production rate did not change significantly. In the group given feed supplemented with 4% valine, the total food intake of the fifth instar was reduced. The body weight of this group was lower than that of the control group (Figure 5A). No significant difference was observed in the total cocoon weight between the different valine groups and the control group (Figure 4B). Valine improved the conversion efficiency of the entire cocoon and reduced the cocoon layer-conversion efficiency. It was speculated that the dry-matter content in the bodies of silkworms in the 4% valine group was higher than that in the bodies of the silkworms in other groups or that there was a problem in water metabolism during silk spinning. More water was retained in the silkworm body. With an increase in the concentration of valine added to the feed, the feed efficiency in the groups with 2% and 4% valine content increased. In contrast, the feed with different valine concentrations showed no difference in digestibility compared to the control group, indicating that the increase in feed efficiency was caused entirely by valine intake. Valine intake could change the balance system of silk pupae, allowing more nutrients to be allocated to them.

Male silkworms were used in this study, so the number of eggs laid could not be calculated. We speculate that the effect on female silkworms is consistent with that on male silkworms, which show increased pupal weight, suggesting that valine can be used as a feed additive to support cocoon production in the silkworm seed-production industry. Based on a production rate 25,000 eggs per silkworm, 4575 g of dry feed powder could be saved by the addition of 4% valine and 1900 g of dry feed powder could be saved by the addition of 2% valine during the fifth-instar period. The 0.5% and 2% valine groups improved the uniformity of the upper cluster; therefore, it is appropriate to add 2% valine to the feed. Feed supplementation with valine causes changes in material metabolism in silkworms, resulting in changes in the retention ratio of nutrients in pupae and in silk protein. Further discussion of these issues will inspire further research into the mechanism of silk–pupa balance.

## 5. Conclusions

In this study, metabolomics analysis revealed that the levels of amino acids in the hemolymph and silk gland of silkworms in the artificial-feed group were significantly lower than those in the hemolymph and silk gland of silkworms in the mulberry-leaf group. Furthermore, we confirmed that valine a is key amino acid in regulating feed efficiency. Our research results provide insights to support further improvement to the formulation of artificial feed for silkworms.

## Figures and Tables

**Table 1:** Differential abundance of cocoon metabolites between the mulberry-leaf and artificial-diet groups.

Metabolite	Category	*p*	AC/MC
urea	Organic acid	3 × 10^−12^	819.98
*D*-pinitol	Sugar	3 × 10^−18^	136.78
glycerol	Alcohol	9 × 10^−13^	66.77
*D*-gluconic acid	Organic acid	7 × 10^−14^	8.61
(R*, S*)-3,4-dihydroxybutanoic acid	Organic acid	2 × 10^−12^	5.96
benzoic acid	Organic acid	4 × 10^−16^	4.88
propanoic acid	Organic acid	7 × 10^−15^	4.36
2,3,4-trihydroxybutyric acid	Organic acid	1 × 10^−6^	3.09
*D*-(+)-talose	Sugar	6 × 10^−3^	1.60
butanedioic acid	Organic acid	1 × 10^−3^	1.22
*L*-threonic acid	Organic acid	2 × 10^−9^	0.42
citric acid	Organic acid	4 × 10^−10^	0.40
*L*-tyrosine	Amino acid	4 × 10^−4^	0.37
2-butenedioic acid(E)	Organic acid	1 × 10^−12^	0.24
phenylalanine	Amino acid	4 × 10^−4^	0.15
*L*-threonine	Amino acid	9 × 10^−5^	0.10
*L*-leucine	Amino acid	5 × 10^−13^	0.10
*L*-isoleucine	Amino acid	1 × 10^−13^	0.07
*L*-valine	Amino acid	1 × 10^−12^	0.05
glycine	Amino acid	2 × 10^−10^	0.05
serine	Amino acid	1 × 10^−10^	0.04
*L*-asparagine	Amino acid	6 × 10^−5^	0.03

AC: cocoons of the artificial-diet group; MC: cocoons of the mulberry-leaf group. *p*: *p*-value. AC/MC: the mean value of AC/the mean value of MC. Data were expressed as mean ± SEM. Differences in data were assessed using Student’s *t*-test (two-tailed, two-sample equal variance hypothesis). MC, *n* = 8 replicates; AC, *n* = 8 replicates.

**Table 2:** Hemolymph metabolites with differential abundance between mulberry leaf and artificial-diet groups.

Metabolite	Category	*p*	AH/MH
*D*-glucopyranoside	Glycosides	2 × 10^−16^	0.06
*L*-valine	Amino acid	2 × 10^−9^	0.08
*L*-leucine	Amino acid	2 × 10^−9^	0.10
butanoic acid	Organic acid	2 × 10^−6^	0.10
2-keto-L-gluconic acid	Organic acid	1 × 10^−10^	0.14
*L*-isoleucine	Amino acid	2 × 10^−7^	0.14
*Myo*-inositol	Sugar	9 × 10^−7^	0.14
methylmaleic acid	Organic acid	2 × 10^−13^	0.23
*DL*-arabinose	Sugar	3 × 10^−12^	0.28
2-hydroxymandelic acid	Organic acid	3 × 10^−7^	0.30
*L*-threonine	Amino acid	2 × 10^−6^	0.32
*L*-alanine	Amino acid	1 × 10^−6^	0.35
*L*-asparagine	Amino acid	4 × 10^−7^	0.42
*D*-mannose	Sugar	1 × 10^−8^	0.44
glycine	Amino acid	3 × 10^−5^	0.48
phenylalanine	Amino acid	7 × 10^−4^	0.49
hexanedioic acid	Organic acid	9 × 10^−8^	0.56
butanedioic acid	Organic acid	3 × 10^−8^	0.58
2-butenedioic acid	Organic acid	2 × 10^−8^	0.59
phosphoric acid	Organic acid	3 × 10^−3^	0.70
serine	Amino acid	3 × 10^−2^	0.74
*L*-proline	Amino acid	1 × 10^−2^	0.81
1,4-butanediamine	Biogenic amine	3 × 10^−4^	1.23
hexadecanoic acid	Fatty acid	3 × 10^−3^	1.40
β-sitosterol	Sterol and lipid	6 × 10^−4^	1.41
glycerol	Alcohol	1 × 10^−3^	1.41
citric acid	Organic acid	6 × 10^−5^	1.46
*D*-(+)-cellobiose	Sugar	2 × 10^−2^	1.55
*D*-(+)-trehalose	Sugar	6 × 10^−10^	1.68
β-alanine	Amino acid	5 × 10^−5^	1.71
*N*,*N*-dimethylglycine	Amino acid	3 × 10^−6^	1.92
urea	Organic acid	1 × 10^−10^	2.08
*D*-sorbitol	Sugar alcohol	3 × 10^−5^	2.13
pentanedioic acid	Organic acid	6 × 10^−9^	2.14
ritalinic acid	Benzene	1 × 10^−9^	2.16
mannitol	Sugar	2 × 10^−7^	2.20
aminomalonic acid	Organic acid	7 × 10^−4^	2.59
pantothenic acid	Vitamin	1 × 10^−13^	4.33

AH: hemolymph of the artificial-diet group; MH: hemolymph of the mulberry-leaf group. *p*: *p*-value. AH/MH: the mean value of AH/the mean value of MH. Data were expressed as mean ± SEM. Differences in data were assessed using Student’s *t*-test (two-tailed, two-sample equal variance hypothesis). MH, *n* = 8 replicates; AH, *n* = 8 replicates.

## Data Availability

The original contributions presented in the study are included in the article/Supplementary Materials, further inquiries can be directed to the corresponding authors.

## Supplementary Materials

The following supporting information can be downloaded at: https://www.mdpi.com/article/10.3390/insects15040291/s1, Figure S1: z-score plot of the analysis of differential metabolites in the cocoon and hemolymph between silkworms reared on mulberry leaves and the artificial diet; Figure S2: Relative Hierarchical cluster analysis and the heatmap of the differential abundance metabolites in the mulberry leaves group and the artificial-diet group.
