# Supplementary material for: Silkworm Hemolymph and Cocoon Metabolomics Reveals Valine Improves Feed Efficiency of Silkworm Artificial Diet
Source: Insects. 2024 Apr 19;15(4):291. doi: 10.3390/insects15040291 (PMC11050563; doi:10.3390/insects15040291)
Supplement: Supplementary file 1 [file insects-15-00291-s001.zip › insects-2945824-supplementary.pdf]

Supplementary materials

Silkworm Hemolymph and Cocoon Metabolomics Reveals Valine Improves Feed Efficiency of Silkworm Artificial Diet

Jinxin Wu[1†], Lingyi Li[2†], Daoyuan Qin[1], Chen Han[1], Yuanlin Liu[1], Guanwang Shen[1*], Ping Zhao[1*]

[1] Integrative Science Center of Germplasm Creation in Western China (Chongqing) Science City, Biological Science Research Center, Southwest University, Chongqing 400715, China.

[2] Westa College, Southwest University, Chongqing 400715, China.

* Correspondence: gwshen@swu.edu.cn; zhaop@swu.edu.cn; Tel./Fax: Zhao, P. +86-023-6825-0885

† These authors contributed equally to this work.

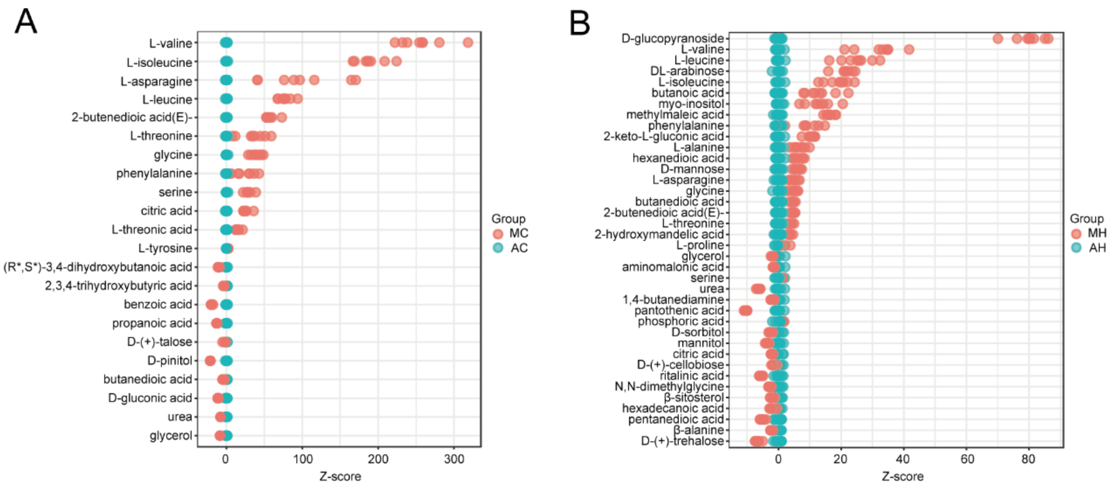

Figure. S1. z-score plot of the analysis of differential metabolites in the cocoon and hemolymph between silkworms reared on mulberry leaves and the artificial diet. (a) cocoon and (b) hemolymph. AC: cocoon of artificial diet rearing group; MC: cocoon of mulberry leaf rearing group. AH: hemolymph of artificial diet rearing group; MH: hemolymph of mulberry leaf rearing group. n=8 replicates.

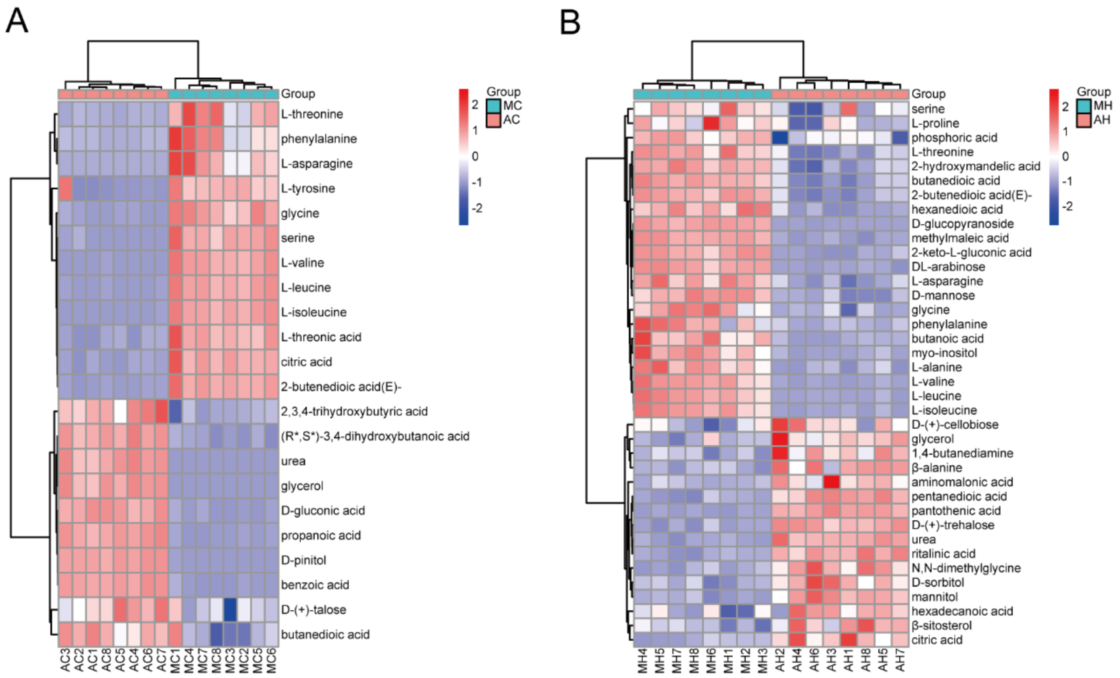

Figure S2. Relative Hierarchical cluster analysis and the heatmap of the differential abundance metabolites in the mulberry leaves group and the artificial-diet group. AC: cocoon of artificial diet rearing group; MC: cocoon of mulberry leaf rearing group. AH: hemolymph of artificial diet rearing group; MH: hemolymph of mulberry leaf rearing group. n=8 replicates.
